# Investigating Early Response to Treatment in a Multi-Site Study for Adolescent Bulimia Nervosa

**DOI:** 10.3389/fpsyt.2020.00092

**Published:** 2020-02-28

**Authors:** Brittany E. Matheson, Sasha Gorrell, Cara Bohon, W. Stewart Agras, Daniel Le Grange, James Lock

**Affiliations:** ^1^ Department of Psychiatry and Behavioral Sciences, Stanford University School of Medicine, Stanford, CA, United States; ^2^ Department of Psychiatry, University of California San Francisco School of Medicine, San Francisco, CA, United States; ^3^ Department of Psychiatry and Behavioral Sciences, Emeritus, Stanford University School of Medicine, Stanford, CA, United States; ^4^ Department of Psychiatry and Behavioral Neuroscience, Emeritus, The University of Chicago, Chicago, IL, United States

**Keywords:** bulimia nervosa, adolescent, treatment, early response, family-based treatment, cognitive behavioral therapy

## Abstract

**Background:**

This secondary data analysis seeks to replicate and extend findings that early response to treatment in adolescent bulimia nervosa (BN) predicts outcome, resulting in earlier identification of patients who might need a different treatment approach.

**Methods:**

Participants were 71 adolescents (*M* ± *SD*: 15.69 ± 1.55 years; 93% female; 75% non-Hispanic) with a Diagnostic and Statistical Manual of Mental Disorders, 4^th^ Edition (DSM-IV) diagnosis of BN or partial BN enrolled in a two-site treatment study. Participants were randomized to cognitive behavioral therapy for adolescents (CBT-A), family-based treatment for BN (FBT-BN), or supportive psychotherapy (SPT). The Eating Disorder Examination was administered at baseline, end-of-treatment (EOT), 6-month, and 12-month follow-up. Binge eating and purge symptoms were self-reported at each session. Outcome was defined as abstinence of binge eating and compensatory behaviors (self-induced vomiting, laxative use, diet pills, diuretics, compensatory exercise, fasting) in the 28 days prior to assessment. Receiver operating characteristic (ROC) analyses were utilized to assess the viability of predicting treatment outcomes based on reduction of symptoms within the first 10 sessions of treatment.

**Results:**

ROC analyses suggest that reduction in purging at session 2 (AUC =.799, *p* < .001) and binge eating at session 4 (AUC =.750, *p* < .01) were independently related to abstinence of symptoms at EOT, regardless of treatment type. Symptom reduction later in treatment predicted outcome at follow-up, as change in binge eating at session 8 and purging at session 9 were the strongest predictors of abstinence at 6-month follow-up (AUCs =.726–.763, *p*s < .01). Change in binge eating, but not purging behaviors, was significantly related to abstinence at 12-month follow-up (AUC =.766, *p* < .01). Only slight differences emerged based on treatment group, such that reductions in symptoms most predictive of abstinence at EOT occurred one session sooner in FBT-BN than SPT.

**Conclusion:**

Reductions in binge eating and purge symptoms early in adolescent BN treatment suggest better outcome, regardless of treatment modality. Additional research with larger samples is needed to better understand which treatments, if any, contribute to earlier change in BN symptoms and/or likelihood of improved patient response.

## Investigating Early Response to Treatment in a Multi-Site Study for Adolescent Bulimia Nervosa

Bulimia nervosa (BN) impacts an estimated 1–3% of adolescents (1, 2), with additional youth meeting partial criteria for the disorder (3, 4). In addition to binge eating episodes and compensatory behaviors (e.g., self-induced vomiting, laxative misuse, excessive compulsive exercise), BN is associated with serious medical consequences, such as electrolyte imbalances and cardiac risks (5, 6), as well as psychiatric comorbidities (7, 8), psychosocial impairment (2), and decreased quality of life (9). Binge eating and compensatory behaviors typically onset during middle to late adolescence (10–13). Thus, targeting intervention efforts in adolescents is critical to address BN symptoms early and prevent chronicity.

Early response to treatment has been studied in adults with BN (14–19) and binge eating disorder (20–23). Specifically, symptom reduction by session 4–8 (week 4) of treatment is predictive of positive outcomes (17, 22). One study found that a 70% reduction in purging by session 6 (4 weeks) of cognitive behavioral therapy (CBT) was related to remission of binge eating and purging symptoms at end-of-treatment (EOT) for adults with BN (14). A separate study found that purging abstinence by session 6 (4 weeks) of treatment (CBT and interpersonal psychotherapy) in adult women with BN was predictive of treatment response at EOT and 8-months post-treatment (15). A 65% reduction in purging by session 8 (4 weeks) of cognitive behavior therapy enhanced (CBT-E) predicted remission at EOT in a sample of adult women with BN (18). In treatment studies for adults with binge eating disorder, reduction of binge eating episodes by 65% at week 4 of treatment (across four medication and psychotherapy treatment conditions) predicted abstinence of binge eating at EOT (21). Thus, symptom change of 65–70% after just 4 weeks of treatment appears to be a critical marker of outcome in adults, regardless of diagnosis or treatment modality.

To date, only four randomized controlled clinical trials have been conducted for the treatment of BN in adolescents (24–27), with one study investigating early treatment response (28). Across both family-based treatment for adolescent bulimia nervosa (FBT-BN) and supportive psychotherapy (SPT) treatment arms, a reduction of binge eating and purging frequency by 85% or more by session 6 (6 weeks) was predictive of remission (defined as the absence of binge eating and purge symptoms in the previous 28 days) at EOT (28). Reductions in symptom frequency by session 6 also predicted remission at 6-month follow-up, such that a ≥ 93% reduction in binge eating and purge symptoms was associated with remission of symptoms (28). Treatment group analyses showed that symptom reduction at session 4 was the strongest predictor of response for the SPT group whereas symptom reduction at session 6 was the strongest predictor for the FBT-BN group (28). Regardless of treatment type, these findings converge with studies suggesting that early treatment response is predictive of improved outcome in adolescents with anorexia nervosa (AN) (29–32).

Given consistent data suggesting that early response to treatment is predictive of improved outcomes in adolescents with AN as well as adults with BN, additional research is needed to assess early treatment response in adolescents with BN and to explore whether early response differs by treatment modality. This secondary data analysis seeks to replicate and extend the findings from Le Grange et al. (28) using a unique data set of adolescents who met Diagnostic and Statistical Manual of Mental Disorders, 4^th^ Edition (DSM-IV) criteria for BN or partial BN. We hypothesize that early treatment response within the first four sessions of treatment will predict abstinence at EOT as well as at 6-months and 12-months post-treatment. We expect time points of early treatment response may differ when analyzing binge eating episodes and purging frequency separately. Results could help to identify patients early in treatment who might benefit from alternative or augmented treatment approaches.

## Method

### Participants

Participants were 71 adolescents with a DSM-IV diagnosis of BN or partial BN (defined as binge eating and purging for more than once/week in last 6 months) enrolled in a two-site treatment study. Participants were randomized to one of three treatment arms: cognitive behavioral therapy for adolescents (CBT-A), family-based treatment for adolescent BN (FBT-BN), or supportive psychotherapy (SPT). Full sample characteristics including treatment outcomes are described in the main outcome report [see (25)]. Participants in this secondary data analysis were a subset of a larger clinical trial (N = 130) for whom weekly binge and purge frequencies during treatment were available (25). Participant data were included if weekly symptoms were recorded for at least 1 of the first 10 treatment sessions. Treatment sessions generally occurred on a weekly basis in the first phase of treatment, with the exception of patient illness, vacations, and other unforeseen absences. Thus, the number of sessions will be reported to account for any disconnect between weeks in treatment and number of sessions delivered. The protocol was approved by the institutional review boards at Stanford University and The University of Chicago. All participants and their parents/guardians completed informed consent procedures and provided written informed assent or consent in accordance with the Declaration of Helsinki.

### Measures

#### The Eating Disorder Examination

The Eating Disorder Examination [EDE; (33)] is a semi-structured interview designed to assess eating disorder behaviors and cognitions with acceptable reliability and validity in treatment-seeking samples (34). The frequency of objective and subjective binge eating episodes as well as purging (self-induced vomiting, laxative misuse, diet pill consumption, diuretics use) and compensatory behaviors (compulsive over-exercise, fasting) were assessed over the previous 28 days. Given that loss of control may be a more salient clinical correlate than episode size (35–37), particularly in younger populations (38, 39), objective and subjective binge eating episodes were summed to calculate total binge eating episodes. The EDE was administered by trained assessors at baseline, EOT, 6-months, and 12-months post-treatment.

#### Binge Eating and Purge Frequency

Participants self-reported the frequency of binge eating and purging episodes over the past week to their study therapist at the start of each session. Participants did not report on additional compensatory behaviors, such as compulsive exercise or fasting, at weekly treatment sessions. Thus, only purging behaviors (self-induced vomiting as well as laxative, diuretic, and diet pill use) were used in calculating reduction of symptoms by treatment session. Study therapists documented these numbers in participants’ study folders.

### Statistical Analysis

Receiver operating characteristic (ROC) analyses were utilized to assess the viability of predicting treatment outcomes based on reduction of symptoms within the first 10 weeks of treatment. Weekly baseline symptom counts were approximated by independently averaging binge eating and purge symptoms over the past 28 days as assessed on the EDE. Participants without reported binge eating or purging behaviors in the past 28 days were not included in relevant analyses. Reductions in baseline binge eating and purge symptoms were calculated relative to these counts for the first 10 sessions of treatment, consistent with prior research (28). Two separate ROC analyses evaluated the relationship between the reductions in binge eating and purge symptoms with treatment outcome at EOT, 6-months, and 12-months post-treatment. Treatment outcome was defined as abstinence from binge eating and compensatory behaviors (self-induced vomiting, laxative use, diet pills, diuretics, excessive exercise, and fasting) in the 28 days prior to assessment, as measured by the EDE. Defining outcome as abstinence of behavioral symptoms is often used as a marker of treatment effect in BN studies (14, 25). Area under the curve (AUC) values were calculated to evaluate the probability that greater reductions in either binge eating and purging symptoms would occur for randomly selected participants that met abstinence criteria compared to those who did not at EOT and follow-up time points. Sensitivity and specificity were also calculated. Results are presented for the entire sample as well as by sub-group analyses based on treatment condition. Analyses were conducted in SPSS version 26 and considered significant at the *p* < .05 level.

## Results

Participants were 71 adolescents between the ages of 12 and 18 years old (*M* ± *SD*: 15.69 ± 1.55 years). The majority of participants were female (93%) and non-Hispanic (75%). Average duration of illness for the sample was a year and a half (*M* ± *SD*: 18.12 ± 17.85 months). The average number of binge eating episodes (objective and subjective binge episodes) over the prior 28 days for the sample at baseline was 22.13 ± 23.17. The average number of purging episodes (self-induced vomiting, laxative use, diet pills, diuretics) over the prior 28 days at baseline was 22.49 ± 22.21. Treatment groups did not statistically differ based on demographic variables or baseline binge eating episodes or purging behaviors (**Table 1**). The average number of treatment sessions completed across the sample was about 14 sessions (*M* ± *SD*: 13.6 ± 5.48 sessions). Number of attended sessions did not significantly differ by treatment group.

**Table 1:** Participant demographics.

	Sample *N* = 71	FBT-BN *N* = 30	CBT-A *N* = 26	SPT *N* = 15	Test Statistic
Age (years)	15.69 ± 1.55	15.87 ± 1.61	15.81 ± 1.65	15.13 ± 1.19	*F* = 1.24, *p* =.230
Sex (female)	66 (93%)	28 (93%)	26 (100%)	12 (80%)	χ^2^ = 5.82, *p* =.054
Ethnicity (% non-Hispanic)	75%	73%	69%	87%	χ^2^ = 1.58, *p* =.455
Race (% Caucasian)	80%	83%	77%	80%	χ^2^ = 6.19, *p* =.626
Duration of illness	18.12 ± 17.85	16.38 ± 20.47	19.00 ± 16.49	20.21 ± 14.82	*F* =.264; *p* =.769
Binge eating episodes	22.13 ± 23.17	26.33 ± 30.06	21.31 ± 16.52	15.13 ± 15.31	*F* = 1.20, *p* =.307
Purge episodes	22.49 ± 22.21	21.10 ± 20.42	25.84 ± 23.79	19.67 ± 23.76	*F* = 0.46; *p* =.635
Session attendance	13.6 ± 5.48	13.96 ± 5.86	14.1 ± 4.87	12.13 ± 6.09	*F* =.709; *p* =.496

FBT-BN, family-based treatment for bulimia nervosa; CBT-A, cognitive behavioral therapy for adolescents; SPT, supportive psychotherapy.

### Early Response Across Treatments

**Figure 1:**
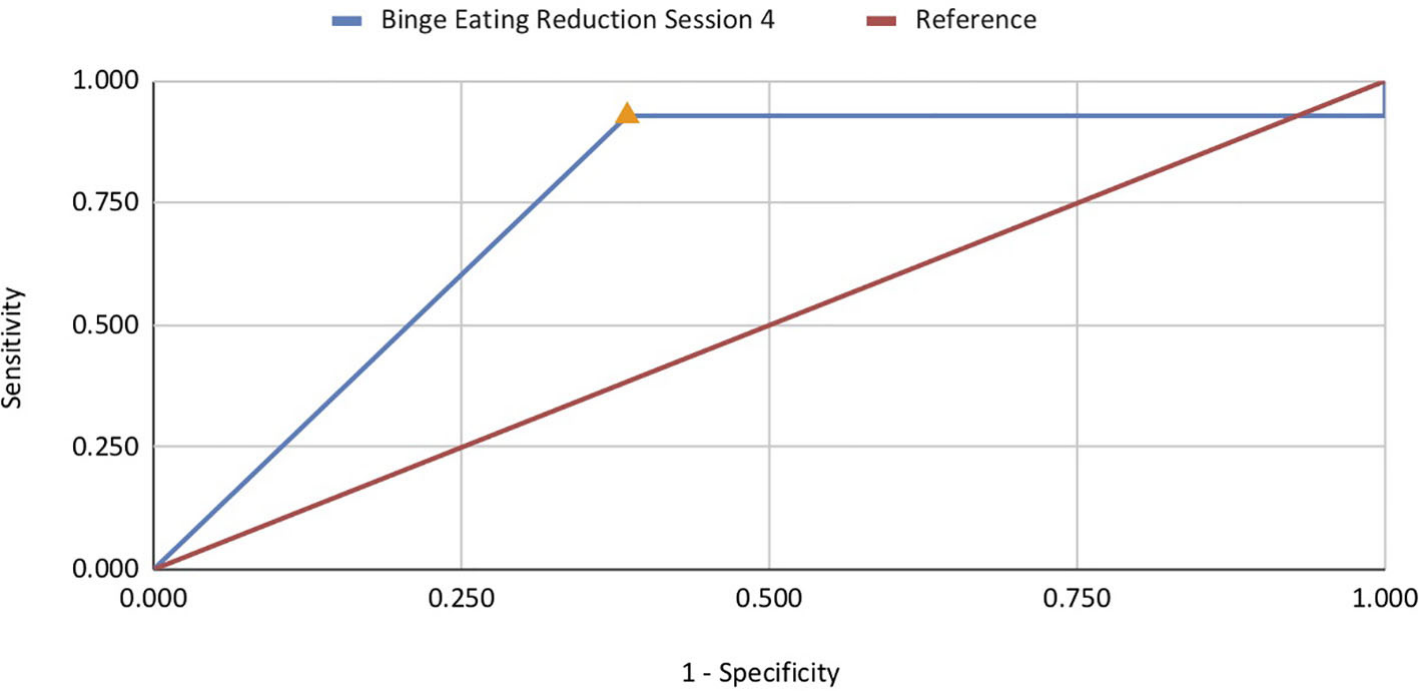
ROC curve for reduction in binge eating predictive of abstinence at end-of-treatment.

**Figure 2:**
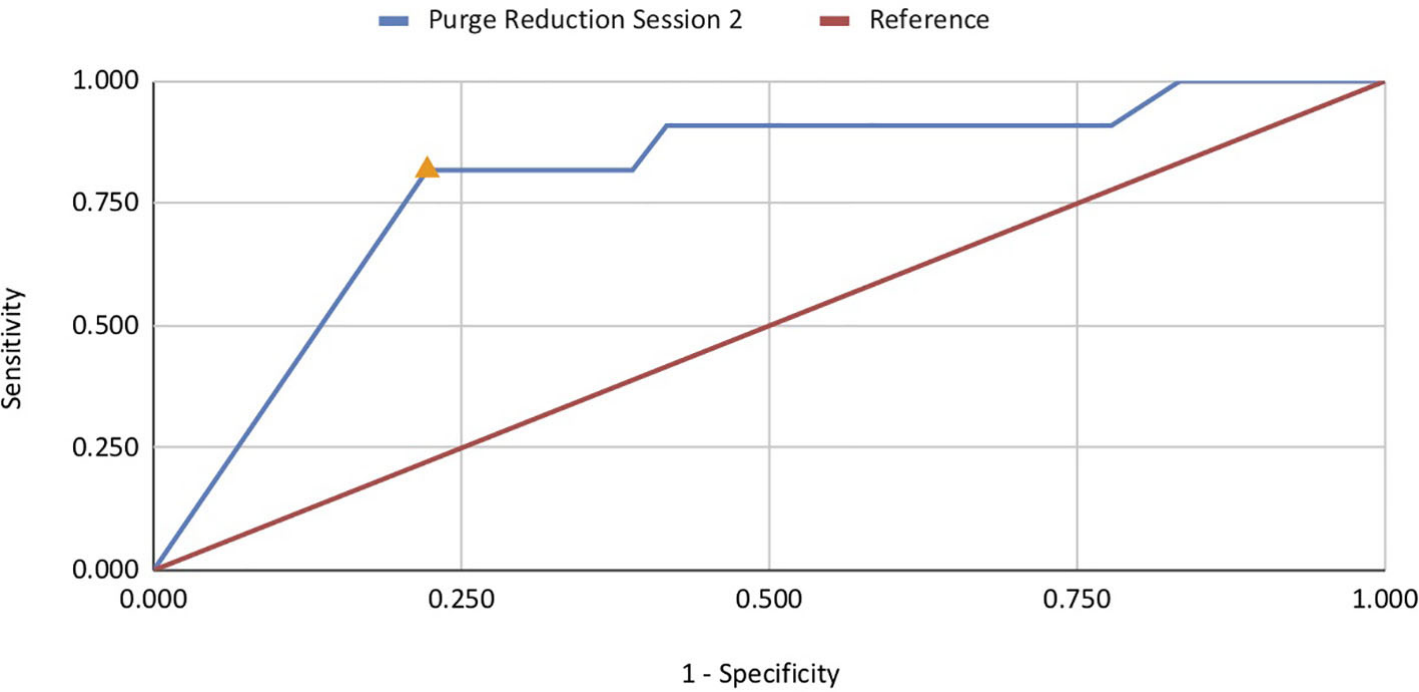
ROC curve for reduction in purging predictive of abstinence at end-of-treatment.

ROC analyses investigated the association between reductions in binge eating and purge symptoms with abstinence at EOT, 6-month, and 12-month follow-up. Reduction in binge eating at sessions 2–5, 9, and 10 was significantly associated with abstinence at EOT, with AUC values ranging from .685 to .750 (**Table 2**). Session 4 was the strongest predictor (AUC =.750, *p* < .01; *n* = 53), with a 96.4% reduction in binge eating by this session achieving optimal sensitivity and specificity (sensitivity =.929; specificity =.615; **Figure 1**). These results indicate that 93% of patients that achieved abstinence at EOT decreased binge eating episodes by at least 96% at session 4 of treatment. Reduction in purge symptoms at sessions 2, 3, and 4 were significantly associated with abstinence at post-treatment, with AUC values ranging from .712 to .799 (**Table 2**). Session 2 was the strongest predictor (AUC =.799, *p* <. 001; *n* = 47), with a 96.8% reduction in purge symptoms by this session achieving optimal sensitivity and specificity (sensitivity =.818; specificity =.778; **Figure 2**). Thus, almost 82% of patients classified as treatment responders showed a decrease in purging episodes by 96.8% at session 2 (**Table 3**).

**Table 2:** AUC for reductions in binge eating and purge symptoms for treatment sessions 1–10.

Session	Binge Eating				Purge			
	N	AUC	SE	CI	N	AUC	SE	CI
**EOT**								
1	58	.563	.091	.385-.741	48	.579	.103	.377–.781
2	57	.716**	.080	.558-.873	47	.799***	.078	.647–.952
3	48	.685*	.091	.507-.863	40	.712*	.093	.529–.894
4	53	.750**	.078	.598-.902	43	.739**	.080	.582–.897
5	45	.688*	.084	.524-.852	37	.615	.104	.412–.818
6	46	.672	.092	.491-.853	40	.644	.099	.450–.839
7	49	.572	.088	.401-.744	39	.600	.102	.401–.799
8	48	.674	.089	.499-.848	40	.648	.099	.454–.843
9	49	.696*	.080	.54-.852	39	.686	.098	.494–.878
10	48	.714	.073	.571-.858	39	.624	.098	.432–.816
**6-month follow-up**								
1	47	.544	.090	.368-.720	38	.386	.106	.178–.595
2	46	.705*	.082	.545-.866	37	.563	.115	.337–.789
3	38	.662	.092	.482-.842	32	.600	.116	.372–.828
4	43	.607	.097	.416-.798	34	.575	.111	.357–.793
5	36	.675	.092	.495-.856	29	.397	.120	.162–.632
6	37	.670	.093	.487-.853	33	.593	.101	.396–.791
7	39	.597	.092	.417-.777	31	.469	.109	.256–.683
8	39	.763**	.075	.616-.909	33	.651	.095	.465–.837
9	41	.750**	.075	.604-.896	33	.726**	.087	.556–.897
10	38	.675	.088	.503-.848	31	.577	.106	.368–.785
**12-month follow-up**								
1	39	.629	.090	.452-.806	30	.397	.108	.186–.609
2	39	.680*	.087	.509-.851	30	.458	.112	.239–.676
3	32	.702*	.097	.512-.893	26	.452	.116	.224–.680
4	36	.583	.097	.392-.774	27	.500	.113	.278–.722
5	31	.679	.097	.489-.868	24	.437	.122	.199–.675
6	31	.575	.106	.368-.782	27	.478	.114	.255–.701
7	33	.656	.096	.468-.845	25	.487	.120	.253–.722
8	32	.689*	.095	.503-.876	26	.509	.116	.282–.736
9	34	.766**	.084	.601-.932	26	.683	.107	.473–.894
10	33	.699*	.095	.513-.884	26	.538	.115	.312–.764

*= p < .05; **= p < .01, ***= p < .001; AUC, area under the curve; CI, confidence interval; EOT, end-of-treatment; SE, standard error.

**Table 3:** Sensitivity and specificity for largest predictors of abstinence by time point.

	Session	AUC	SE	CI	% Reduction	Sensitivity	Specificity
**EOT**							
Purge	2	.799***	.078	.647–.952	96.8%	.818	.778
Binge eating	4	.750**	.078	.598–.902	96.4%	.929	.615
**6-month**							
Purge	9	.726**	.087	.556–.897	94.4%	.833	.571
Binge eating	8	.763***	.075	.616–.909	96.4%	.857	.600
**12-month**							
Purge	9	.683	.107	.473–.894	—	—	—
Binge eating	9	.766**	.084	.601–.932	96.4%	.929	.615

**= p < .01, ***= p < .001; AUC, area under the curve; CI, confidence interval; SE, standard error.

Symptom reduction later in treatment predicted abstinence at follow-up, with reductions in binge eating at session 8 (AUC =.763, *p* < .001; *n* = 39) and reductions in purging at session 9 (AUC =.726, *p* < .05; *n* = 33) predictive of abstinence at 6-month follow-up. Optimal sensitivity and specificity were obtained for a 96.4% reduction in binge eating (sensitivity =.857; specificity =.600) and a 94.4% reduction in purge symptoms (sensitivity =.833; specificity =.571). A decrease in binge eating but not purging episodes at session 9 was predictive of abstinence at 12-month follow-up (AUC =.766, *p* < .01; *n* = 34), with a 96.4% reduction in symptoms predicting outcome (sensitivity =.929; specificity =.615). Reduction in purging behaviors during sessions 1–10 did not significantly predict abstinence at 12-month follow-up (*p* > .05).

### Early Response Differences by Treatment Group

Slight differences emerged based on treatment group, such that reduction in purging at session 2 in FBT-BN (AUC =.754, *p* < .05) and sessions 3 and 4 in SPT (AUC =.917, *p* < .001) were the strongest predictors of abstinence at EOT (**Table 4**). CBT-A demonstrated poor model quality for significant results, due to small sample size with few responders at EOT. Group differences occurred later for binge eating, with reductions by session 4 in FBT-BN (AUC =.767, *p* < .01) and sessions 5 and 6 in SPT (AUC =.833, *p*s < .05) most predictive of treatment response. Although session 5 in CBT-A demonstrated the greatest AUC values for binge eating, the results were not significant (AUC =.773, *p > .*05). Small sample size and missing data precluded meaningful sub-group analyses for follow-up time points.

**Table 4:** Treatment group differences in AUC for reductions in binge eating and purge symptoms for treatment sessions 1-10 predicting abstinence at end-of-treatment.

	FBT-BN				CBT-A				SPT			
	N	AUC	SE	CI	N	AUC	SE	CI	N	AUC	SE	CI
**Binge eating**												
1	27	.465	.122	.226-.703	18	.563	.239	.094–1.031	13	.750	.139	.477–1.023
2	27	.700	.105	.494-.906	18	.453	.324	-.182–.906	12	.722	.152	.425–1.02
3	23	.662	.118	.431-.892	15	.423	.308	-.180–1.026	10	.813*	.142	.533–1.092
4	26	.767**	.096	.579-.955	18	.422	.306	-.178–1.021	11	.813*	.131	.555–1.07
5	24	.611	.119	.377-.844	12	.773	.178	.424–1.121	9	.833*	.140	.559–1.107
6	23	.658	.119	.424-.891	15	.442	.275	.834–.982	8	.833*	.150	.540–1.127
7	23	.569	.121	.332-.807	16	.304	.251	-.189–.796	10	.813*	.142	.533–1.092
8	23	.615	.122	.377-.854	16	.767	.170	.434–1.100	9	.813	.171	.477–1.148
9	23	.658	.119	.424-.891	16	.750	.142	.472–1.028	10	.750	.165	.426–1.074
10	23	.654	.114	.429-.878	16	.750	.142	.472–1.028	9	.750	.204	.350–1.150
**Purge**												
1	22	.513	.137	.246-.781	15	.464	.135	.200–.728	11	.556	.261	.045–1.066
2	22	.754*	.107	.545-.964	15	.214	.110	-.001–.429	10	.875**	.116	.649–1.101
3	20	.635	.127	.387-.884	12	.318	.143	.039–.598	8	.917***	.107	.708–1.126
4	20	.654	.123	.413-.894	15	.393	.132	.134–.652	8	.917***	.107	.708–1.126
5	20	.648	.131	.391-.905	11	—	—	—	6	.750	.210	.338–1.162
6	19	.648	.131	.391-.905	12	.167	.108	-.044–.378	8	.750	.182	.394–1.106
7	19	.659	.128	.409-.909	13	.250	.125	.005–.495	7	.667	.261	.156–1.177
8	19	.608	.133	.347-.869	14	.769	.173	.430–1.109	7	.667	.261	.156–1.177
9	19	.648	.131	.391-.905	13	.792	.164	.470–1.114	7	.750	.220	.318–1.182
10	19	.568	.135	.303-.833	13	.792	.164	.470–1.114	7	.333	.192	-.044–.771

*= p < .05; **= p < .01, ***= p < .001; AUC, area under the curve; CBT-A, cognitive behavioral therapy for adolescents; CI, confidence interval; FBT-BN, family-based treatment for bulimia nervosa; SE, standard error; SPT, supportive psychotherapy.

## Discussion

Significant reductions in binge eating and purge symptoms earlier in treatment suggest better outcome for adolescents with BN at EOT, regardless of treatment modality. This study demonstrated that reductions ≥ 96.8% in purging episodes by session 2 and reductions ≥ 96.4% in binge eating episodes by session 4 predict abstinence at EOT. Additionally, this study sought to better understand differences in symptom change for binge eating and purge behaviors separately, suggesting that an earlier reduction in purging behaviors compared to binge eating was related to treatment response at EOT. The only prior study of early response to treatment for adolescent BN collapsed across binge eating and purging symptoms (28), while Nazar et al. (17) in their review note that adult studies reported purging reduction only. Thus, it is unknown if reductions in behavioral symptoms, i.e., binge eating or purging, which are indicative of early response, occur at similar or differential rates. It may be important to assess for and evaluate early reductions in these behaviors separately in order to better predict early treatment response. The results of this study also found an earlier treatment response than the only prior study in adolescent BN, which reported symptom change by session 6 predicted outcome at EOT (28). In contrast to previous findings, reductions in binge eating and purging later in treatment (session 8 and session 9, respectively) as opposed to session 6 (28), were related to abstinence at 6-month follow-up. Similarly, binge eating, but not purge, reductions at session 9 were predictive of abstinence at 12-month follow-up in the current study. Given the limited data available for the 6-month and 12-month time points, results should be interpreted cautiously. Additional research should be conducted to understand what relationship, if any, early treatment response has in predicting abstinence over time.

These preliminary results indicate that change in symptom reduction suggestive of a positive outcome occurs at similar time points in treatment across modalities. Specifically, abstinence at EOT is predicted by change in behavior one session sooner for both binge eating and purging frequency in FBT-BN compared to SPT. It is possible that the direct focus on symptom reduction in behaviorally-focused treatments or the involvement of family members in disrupting eating disorder behaviors contributes to this slightly earlier change. However, the cell size for each treatment was quite small (*n* = 8–26) and thus research with larger-sized treatment arms is needed. Future research should investigate treatment-based differences in early symptom reduction predictive of response status. Additional research into treatment-specific early behavioral response could help develop clinical benchmarks to guide treatment and assess progress.

Importantly, identifying behavioral markers predictive of treatment response could signal the need for adaptations or different treatment approaches earlier in treatment for individuals likely not to respond (32). Research suggesting the importance of early weight gain (2.4 kg by session 4) in FBT for adolescent AN led to the development of treatment adaptations for likely non-responders in an effort to improve outcome (40, 41). Additional studies across eating disorder diagnoses are needed to assess whether supplementing or shifting treatment course improves outcomes for individuals identified as not likely to respond based on failure to achieve early behavioral change. Further, clinicians may consider assessing change in symptoms during early treatment sessions and re-evaluate by session 4 if lack of improvement noted. It may also be useful for clinicians to examine changes in binge eating and purging episodes separately if future research confirms purge reduction occurs earlier than binge eating reduction for patients who may be most responsive to treatment.

This study, as well as the Le Grange et al. (28) study, showed larger percent reductions in binge eating and purging episodes early in treatment for adolescents compared to studies in adults [82–97% compared to 45–70%; (17)]. Differences in study design and definition of treatment response may account for variability in symptom reduction. Further, these observed age-based differences could also be related to duration of illness, as adolescents on average may not have suffered as long as adults from BN symptoms. Given that shorter duration of illness is related to improved treatment response for eating disorders as well as other psychiatric disorders (42–44), it is possible that adolescents may be more responsive to treatment and therefore show earlier reductions in symptoms compared to adults. Future research should seek to better understand differences between youth and adults on markers of early treatment response and clinical improvement.

There are several limitations of the current study, including missing data and small sample size, particularly in sub-group analyses. Participants self-reported binge eating episodes and purging frequency to their therapist each session, and as such, accuracy may be limited by recall difficulties or presentation bias. The use of weekly monitoring logs or ecological momentary assessment strategies may help combat these limitations in future studies (45). Additionally, the sample was largely female, non-Hispanic, and Caucasian, and thus findings may not generalize to more diverse populations. Further, purging behaviors reported at treatment sessions were not separated by type. Thus, it is unknown if different rates of change occurred based on purging behavior or whether certain purging behaviors, such as laxative use, were more resistant to early change. Moreover, participants only reported purging episodes and thus reductions in additional compensatory behaviors, such as excessive exercise and fasting, are not captured in this study. Future research should seek to collect information on various compensatory behavior strategies separately to further identify whether reductions differ by type of behavior.

Research investigating treatment outcomes for adolescents with BN is lacking. As such, this study sought to better understand behavioral markers of early treatment response within this population. This study also provides additional evidence that clinical improvements in behavioral symptoms within the first four sessions of treatment is indicative of treatment response. Interestingly, reductions in purging episodes occurred before binge eating in this sample, which suggests that researchers and clinicians may want to evaluate these behavioral symptoms separately when ascertaining likelihood of treatment response. These data also suggest that routine assessment of symptom change early in treatment could help providers decide to augment or switch modalities if significant reductions in behavioral symptoms are lacking by session four. Future research should continue to investigate the relationship among early symptom change and treatment outcomes to better inform clinical care for adolescents with BN.

## Data Availability Statement

The datasets generated for this study are available on request to the corresponding author.

## Ethics Statement

The protocol was approved by the Institutional Review Boards at Stanford University and The University of Chicago. All participants and their parents/guardians completed informed consent procedures and provided written informed assent or consent in accordance with the Declaration of Helsinki.

## Author Contributions

All authors contributed to the conception and design of this study. DG, JL, and WA designed and implemented the randomized controlled trial from which this secondary data analysis resulted. All authors contributed to the writing, editing, and revision of this manuscript and approved the final version.

## Funding

SG is supported by the National Institutes of Health (T32 grant MH0118261-33); R01-MH-079979 (DG); and R01-MH-079980 (JL).

## Conflict of Interest

DG receives royalties from Guildford Press and Routledge. He also is co-director of the Training Institute for Child and Adolescent Eating Disorders, LLC. JL receives royalties from Guildford Press. He also is co-director of the Training Institute for Child and Adolescent Eating Disorders, LLC.

The remaining authors declare that the research was conducted in the absence of any commercial or financial relationships that could be construed as a potential conflict of interest.
